# What is in the Word “Antibiotic?” From Microbial Warfare to Microbial Signaling

**DOI:** 10.1093/ofid/ofag632

**Published:** 2026-10-13

**Authors:** Carlos R V Kiffer

**Affiliations:** Infectious Diseases Discipline, Grupo de Análises em Infecções e Antimicrobianos (GAIA, Laboratório Especial de Microbiologia Clínica – LEMC), Antimicrobial Resistance Institute of São Paulo (ARIES), Escola Paulista de Medicina (EPM), Universidade Federal de São Paulo – UNIFESP, São Paulo, Brazil

To the editor—We read with great interest the recent review by Beisel et al on the etymology and history of antibacterials and antifungals. Their account illustrates how the history of antimicrobial therapy has been accompanied by a powerful vocabulary of conflict. Paul Ehrlich's magic bullet anticipated the idea of selectively attacking a pathogen, while the term antibiotic, introduced by Selman Waksman in 1941, came to describe microbial substances capable of inhibiting or killing other microorganisms. The authors appropriately note how this history ultimately contributed to the familiar metaphor of an antimicrobial “arms race” [1].

We suggest that this terminology deserves consideration not only from an etymological perspective, but also from a cognitive one. Could the words and metaphors through which we conceptualize antimicrobial therapy subtly influence how clinicians and patients think about microorganisms and infection?

Contemporary experimental cognitive science studies suggest that linguistic categories can direct attention toward particular features of experience and influence perceptual discrimination, categorization, memory, and other cognitive processes [2, 3]. In this sense, language could be understood as one of several mechanisms that repeatedly train attention toward particular ways of partitioning it. Seen through this lens, antibiotics are an interesting word and example for medicine and science in general. Its conceptual history privileges antagonism, and maybe a sense of bellicism against microorganism. It means that there is a microorganism, an agent directed against it, and an expected inhibition or elimination. On the one hand, despite the words or languages used to name these agents, this framework has been enormously productive and has generated uncountable therapeutic advances. On the other, it is possible that the exact same language has fostered and encouraged an overly simplified representation of microbial ecology, ie, the notion that successful treatment consists almost exclusively of selecting an increasingly powerful “weapon” against an identifiable “enemy.”

Microbiology itself offers reasons to question that representation. It has already been two decades since Linares et al proposed a quite interesting term for antimicrobials: “Antibiotics as intermicrobial signaling agents instead of weapons” [4]. In this stimulating article, they demonstrated that subinhibitory concentrations of several antibiotics altered gene expression and phenotypes in *Pseudomonas aeruginosa*, including biofilm formation, motility, and virulence-related functions [4]. Rather than viewing antibiotics exclusively as weapons used by microorganisms against competitors, as their title proposes, they suggested that, at concentrations encountered in natural environments, these molecules may also function as intermicrobial signals contributing to the regulation of microbial communities. Their experiments thus supported a conceptual shift from microbial weapons toward collective regulators of microbial homeostasis [4].

Beisel et al [1] paper made me wonder if the distinction is more than semantic. Molecules that humans administer at sufficiently high concentrations to inhibit or kill bacteria may, in evolutionary, ecological and occasionally in clinical contexts, participate in biological interactions considerably more complex than warfare. We appropriated components of an ancient microbial chemical language and transformed them into extraordinarily effective medicines. And, by doing so, the vocabulary of the antibiotic era may have inadvertently reinforced a degree of therapeutic hubris. Clinical expressions such as “stronger antibiotics,” “broadening coverage,” and “fighting resistance” remain useful shorthand, but collectively they may sustain the impression that microbial problems are solved primarily through escalation of antimicrobial force. Antimicrobial resistance provides a powerful reminder that microorganisms are not passive targets, but rather are evolving populations embedded within complex ecosystems.

I am not proposing that the terms antibiotic or antimicrobials should be totally abandoned, nor that linguistic framing has been demonstrated to cause inappropriate antimicrobial prescribing. That causal claim would exceed the current letter and the available evidence. Rather, the historical perspective offered by Beisel et al [1] invites a broader question: does the language with which we describe antimicrobials also shape the conceptual models through which generations of clinicians learn to use them?

Perhaps, the next chapter in antimicrobial stewardship requires not only better drugs and better prescribing, but also a measure of linguistic humility. Antimicrobials remain lifesaving tools when appropriately deployed. But they may also be molecules borrowed from a microbial conversation that began billions of years before we learned how to listen and, even, how to speak about them.
